# A Community-in-the-Loop Approach to Smart Home Monitoring for Aging in Place: Mixed Methods Evaluation of a Co-Designed Prototype

**DOI:** 10.2196/88290

**Published:** 2026-07-23

**Authors:** Roschelle L Fritz, Connie Kim Yen Nguyen-Truong, Jennifer Phipps, Ellen E Hinderlie, Yolanda LB Rodriguez, Thai Hien Nguyen, Gabrielle Barling, Anna Mishuk, Heather Schoonover, Christi Zuber, Marilyn Rantz

**Affiliations:** 1 Faculty School of Nursing, UC Davis Health System, 2570 48th St., Sacramento, CA, 95817, United States, 1 916-734-4349; 2 Department of Nursing and Systems Science, Faculty College of Nursing, Washington State University Vancouver, Vancouver, WA, United States; 3 School of Nursing, University of California Davis Health, Sacramento, CA, United States; 4 College of Nursing, Washington State University Vancouver, Vancouver, WA, United States; 5 Advanced Practice & Community-Based Care Department, Faculty College of Nursing, Washington State University Vancouver, Vancouver, WA, United States; 6 Community Health Worker, Vancouver, WA, United States; 7 Amazon (United States), San Diego, CA, United States; 8 Health Catalyst, South Jordan, UT, United States; 9 Aspen Labs, Denver, CO, United States; 10 Sinclair School of Nursing, University of Missouri, Columbia, MO, United States

**Keywords:** smart homes, ambient sensors, health equity, remote patient monitoring, aging in place, community-based participatory research, co-design, digital distress

## Abstract

**Background:**

The population of adults aged 65 and older is rapidly increasing, while the availability of caregivers is declining. Smart homes that provide unobtrusive, continuous monitoring and alerting on clinically relevant changes in daily activity patterns offer a potentially innovative solution for aging in place.

**Objective:**

This study aims to evaluate the barriers and facilitators to the adoption of a low-cost smart home embedded within a community-based approach to health monitoring for older adults with multiple chronic conditions and who are experiencing poverty.

**Methods:**

Using a prospective, mixed methods design and iterative community co-design, 46 older adults from 7 different language groups were continuously monitored for 6 months with ambient sensors installed in their homes. Two older adults were monitored for 4 and 5 months, respectively, resulting in a total sample of 48. The system generated alerts based on movement pattern changes and escalated notifications to participants, support persons, community health workers, and nurses. Sensor data were analyzed descriptively to quantify alert patterns and response rates, while written text-based data from in-the-moment surveys, community health workers’ and registered nurses’ notes, and semistructured interviews underwent qualitative descriptive analysis and reflexive thematic coding.

**Results:**

The system generated 37 million sensor readings condensed into 1.2 million high-level events and 4719 novel alerts. Qualitative data comprised 34,086 words of text. Participants responded to 1.57% (74) of the initial email alerts and 7.79% (368) of the follow-up SMS text message alerts sent when no email response was received. Community health workers and registered nurses responded to 78.36% (n=3698) of the escalated alerts, resulting in 1060 contacts with participants in response to alerts. Clinical contacts resulted in 72 interventions. Three major qualitative themes emerged: (1) Alone, (2) Trust, and (3) Human Connection. Subthemes included Safety, Personalization, and Digital Distress defined as stress associated with interacting with digital health-monitoring systems. Participants rated the system highly (mean likelihood-to-recommend rating 8.68/10, SD 1.68); however, they expressed a strong preference for phone calls over automated alerts. Cultural expectations influenced adoption, particularly in multigenerational households.

**Conclusions:**

Communities can effectively engage in technology-delivered health care. Future research is needed to improve technical aspects of smart home monitoring systems, including accurate alerting using machine learning, data visualizations for older adults and health care workers, and culturally sensitive features. Additional work should address how and when to communicate automated messaging, engage older adults with their own data, and integrate sensor-based monitoring into health care workflows. Research should also explore personalization through advanced computational approaches such as machine learning and strategies to reduce digital distress.

## Introduction

### Background

The global population is aging rapidly, with an estimated 1.6 billion adults aged 65 and over by 2050 [1]. This demographic shift is accompanied by a shrinking caregiver-to-older-adult ratio: in the United States, the ratio for adults aged 80 and older is projected to decline from 7:1 in 2010 to 3:1 by 2050, with fewer caregivers available for the growing aging population [2,3]. This imbalance presents the urgent challenge of how to support older adults in aging safely and independently at home.

Health monitoring smart homes (ie, sensor-based health monitoring at home) offer a promising solution [4,5]. These systems typically consist of ambient sensors (eg, motion, door, humidity, and more) connected to a central hub that transmits data to a secure platform. Algorithms analyze movement patterns to detect anomalies such as prolonged inactivity, disrupted sleep, or changes in kitchen or bathroom use, all potential indicators of emerging health concerns. Alerts can be communicated via email, SMS text message, or phone calls, and escalated to caregivers or clinical teams for follow-up.

However, most smart home health monitoring has been developed and tested in higher-income settings, leaving critical gaps in understanding their feasibility and acceptability among older adults experiencing poverty [6,7]. Older adults in subsidized housing or with limited income face distinctive barriers to technology adoption, including limited internet access, low digital literacy, mistrust of systems, and cultural expectations around caregiving [8-12]. Without deliberate attention to these barriers, smart home innovations may risk widening health disparities rather than closing them [13,14].

This study aims to identify and understand barriers to adopting smart home health monitoring occurring within a co-designed community support system among older adults experiencing poverty. To address these aims, a low-cost, community-in-the-loop “smart health system” prototype was developed, featuring continuous ambient health monitoring capabilities. Using mixed methods, we evaluated how older adults interacted with the system, the challenges they encountered, and the features that supported trust, engagement, and equitable access. Here, the term *smart home* refers broadly to ambient sensing in the home for the purpose of monitoring residents’ health. The prototype deployed for this study is referred to throughout as a *smart health system* because the smart home was embedded in a *community-in-the-loop* support system where older adults received a smart home along with systematic support from self-identified family and friends (ie, a support person) and from appointed community health workers (CHWs) and registered nurses (RNs).

This paper shares the findings from trialing the community-in-the-loop approach to health monitoring in resource-constrained settings that use smart homes and discusses the potential for smart home adoption by older adults living in poverty. The purpose of this paper is to provide insights into how, and if, continuous ambient health monitoring is possible in older adult populations experiencing poverty. The low-cost system is discussed in detail in Multimedia Appendix 1.

### Smart Home Technologies for Health Monitoring

Smart home systems leverage ambient sensors, Internet of Things (IoT) devices, and data analytics to monitor daily activities and detect health-related changes [14-16]. Prior studies demonstrate their potential to support aging in place by identifying deviations in mobility, sleep, and self-care routines. Rantz et al [17,18] showed that sensor-based systems can detect early signs of health decline. Pioneering work by Cook and the team at the Center for Advanced Studies in Adaptive Systems (CASAS) developed lightweight, easy-to-install systems for activity recognition and health monitoring, including nurse-in-the-loop models and pain-related behavior detection [5,19-22]. More recent work has advanced IoT-based frameworks for secure remote monitoring [23] and patient-centric agents using blockchain on 5G networks [24]. Furthermore, work has been done that specifically identifies gaps in chronic disease applications [4].

Smart home systems show promise in detecting early signs of clinically relevant conditions such as cardiovascular disease, congestive heart failure, cancer, urinary tract infections, sleep disturbances, and cognitive decline [4,21,25]. These conditions disproportionately affect older adults experiencing poverty who often face delayed diagnoses and limited access to care [26-29]. Sensor-based alerts, such as changes in mobility, sleep patterns, or hygiene routines, can serve as proxies for emerging health issues and prompt timely interventions. These studies confirm the feasibility of unobtrusive monitoring but often remain focused on higher-income populations.

### Barriers and Facilitators to Adoption in Low-Income Populations

While smart home technologies are well established, their adoption among diverse older adults remains understudied. Prior work has primarily focused on for-profit senior living settings, including Tiger Place [17,18], Touchmark [21,30,31], and early collaborative aging research using technology (CART)–related studies [32] where adoption has not faced many barriers. In contrast, adoption barriers among low-income older adults persist, including privacy concerns, perceived intrusiveness, system complexity, digital literacy challenges, limited internet access, and inadequate technical support [4,7,33-35]. Similar inequities appear across digital health interventions, such as mobile health and telehealth [36], for chronic disease management [37-39].

Key facilitators for older adults and the health care team include ease of use, perceived usefulness, and a system’s ability to enhance safety and independence [4,9,40-42]. Trust in the technology and the organizations deploying it strongly shapes adoption, particularly amid concerns about data security and reliability [4,9,43]. This study extends prior work by examining barriers and facilitators to adoption among older adults with multiple chronic conditions living in poverty, with particular attention to affordability, usability, cultural relevance, and community co-design as key determinants of trust and engagement.

### Community Engagement and Co-Design

Co-design and community-based participatory research methodologies have proven effective in tailoring digital health interventions to the needs of diverse populations [44,45]. Involving end users in design and testing enhances trust, usability, and perceived ownership of technology [46,47]. However, few studies have applied these principles to smart home health monitoring systems serving low-income older adults with multiple chronic conditions.

This study embedded community engagement across all phases of system development and evaluation [48]. We brought together low-income older adult residents in subsidized housing and living in the community, subsidized housing managers, CHWs from diverse cultural and ethnic communities, and nursing and engineering researchers to co-design features including notification preferences, sensor placement, and privacy safeguards. Adapted from the “nurse-in-the-loop” model, this *community-in-the-loop approach* ensured alignment with participants’ cultural, social, and socioeconomic contexts. Iterative feedback revealed preferences for low-burden devices, clear feedback loops, and direct human contact during alerts, showing how co-design can make smart home health systems more inclusive, acceptable, and scalable in resource-constrained settings.

## Methods

### Ethical Considerations

This study was approved by the Washington State University Institutional Review Board (IRB) for human participants’ research under IRB#19135. All participants provided informed consent in their preferred language. Seven languages were represented in the participant pool. Each participant received a US $50 gift card and a results summary after study completion.

### Study Design and Setting

A community-engaged, prospective, mixed methods design was used to prototype and evaluate a low-cost smart health monitoring system for older adults managing multiple chronic conditions while experiencing poverty. The study was conducted in collaboration with local housing authorities, culturally based health organizations, and a free community health clinic in the US Pacific Northwest.

### Smart Health System Prototype

**Figure 1.**
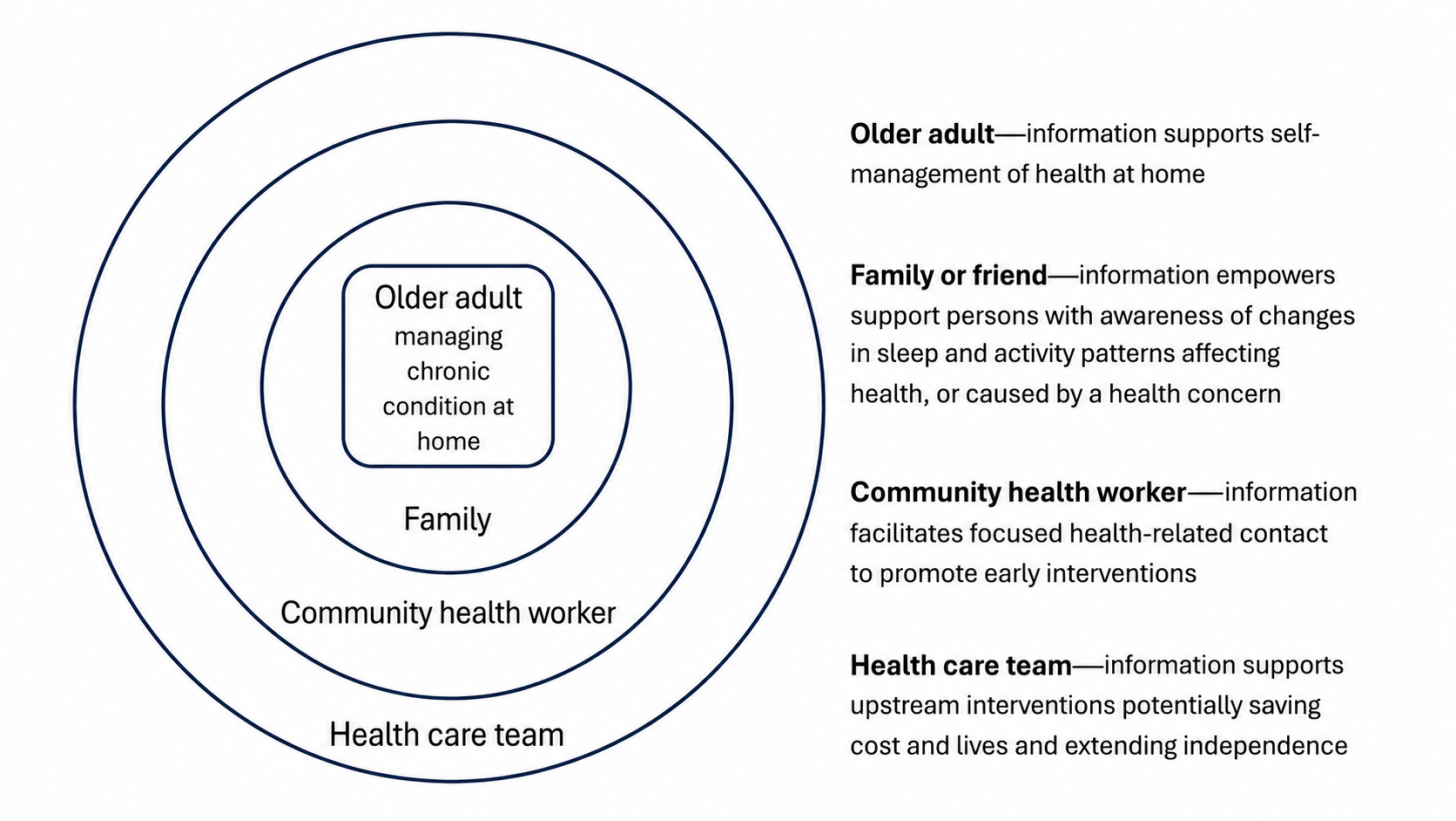
The connected smart health system.

The prototype system was designed as a closed-loop model to help families, communities, and health care teams support older adults aging in the place of their choosing (Figure 1). The system operated through three integrated components: (1) ambient sensing, (2) a rules-based algorithm developed for this study that continuously processed sensor data and automatically generated alerts for clinically relevant changes in daily behavior, and (3) community members who worked alongside the technology to respond promptly to alerts, including facilitating engagement with the health care team.

The prototype system included 6 alert types, with alert messaging designed not to imply judgment or provoke stress or fear. The system alerted for social isolation (Homebody), decreased activities (Daytime Resting), kitchen use (Eating Routines), decreased hygiene (Bathing), sleep disturbance (Restless Night), and sleep patterns (Changing Sleep Routine). The participant-facing alert terms associated with clinically relevant behavior changes are italicized. Multimedia Appendix 1 contains detailed descriptions of alerts, including defined thresholds.

### Study Sample and Recruiting

A convenience sample was recruited with the assistance of subsidized housing building managers. More than 80 residents across 7 subsidized housing properties attended information presentations about the smart health system, after which interested individuals met privately with a trained research assistant or the lead primary investigator (PI) to review consent forms in their preferred language. Residents were informed that the system was a research prototype, that it was not intended for emergency detection or intervention, and that the absence of alerts would not imply health or clinical stability. They were instructed to use usual health care and emergency services. Adults aged 50 years and above with 2 or more chronic conditions and income at or below the federal poverty level (US $14,580 annually for a single-person household) [49] were included. The lower age threshold was informed by community partner input, which emphasized that chronic conditions often develop earlier among racially and ethnically diverse populations. Older adults living alone were prioritized for recruitment, although homes with fewer than 2 residents met inclusion criteria. A target was set for recruiting >80% single-resident homes. Exclusion criteria included dementia, severe mental illness, or pets that could interfere with sensor readings.

### Data Collection

Data collection combined continuous, ambient, home-based sensing with participant-facing and team-documented narratives, and in-the-moment surveys associated with health alerts. Table 1 contains the data elements of the study with descriptions. Alerts were triggered when anomalies exceeded preset thresholds (eg, no door use for 48 hours, reduced kitchen activity, sleep disruption). To minimize alert fatigue, some overlapping alerts were combined into a single alert, yielding 4719 novel alerts. For example, a Restless Night alert and a Changing Sleep Routine alert on the same night would be combined into one alert requiring one survey response. Because it was not possible to have continuous daily CHW coverage for the study duration, the full-time research RN or lead investigator (an RN) assisted in responding to alerts, and alert notifications were delivered only during business hours. Business hours were 9:00 AM to 6:00 PM Pacific Time, Monday to Friday. Nighttime sensing resulting in alerts saw the alerts delivered at 9:00 AM on weekdays, and weekend alerts were delivered at 9:00 AM on Mondays. No alerts were discarded.

**Table 1.** Data elements with descriptions.

Data element	Description
Sensor data from 6 multisensors per home	The sensor state (ON) when motion was sensed, or when temperature, humidity, and lights changed.
Alerts	Emails and SMS text messages generated by the system and sent to the participant, a support person, and a CHW^a^ (sequentially escalating) when a change in daily routine met algorithm rules aligned with possible health changes.
Alert responses	Discrete and unstructured written text provided by participants, a support person, CHWs, or an RN^b^.
Interview transcripts	End-of-study interviews about acceptance perceptions.
Likelihood-to-recommend rating	Participant rating on a 0‐10 scale indicating how likely they would be to recommend the Smart Health System to family or friends when a system (like the prototype) would be available on the market. Ratings were subsequently categorized as promoters (9-10), passives (7-8), and detractors (0‐6) to calculate the Net Promoter Score.

a CHW: community health worker.

b RN: registered nurse.

Each alert included a secure survey link for reporting current health status. Unacknowledged alerts prompted CHW follow-up and escalation to an RN as needed. Narrative data were drawn from three sources: free-text survey responses, CHW/RN field notes after alert-related contacts, and semistructured exit interviews. Although 6 additional languages (Cantonese, Mandarin, Korean, Vietnamese, Spanish, and Russian) were spoken beyond English, multilingual CHWs communicating in preferred languages documented all responses in English.

At a conceptual level, the smart health system generated alerts using 3 components: sensing, rule-based interpretation, and filtering. Ambient motion and door sensors continuously captured movement throughout the home, and these raw signals were aggregated into higher-level behavioral events representing where and when a participant was active (eg, time in the kitchen, nighttime location, frequency of door use). A set of rules, co-designed with community partners and the PIs who were RNs, compared each participant’s recent behavior against their own established baseline routine to identify clinically meaningful deviations, such as prolonged inactivity, disrupted sleep, reduced kitchen use, or missed bathing patterns. When a deviation crossed a predefined threshold, an alert was generated. To prevent alert fatigue, repeat alerts of the same type within a 1-week window were suppressed.

Multimedia Appendix 1 describes the full technical specifications of the sensors, processing pipeline, rule definitions, and figures exhibiting the health-monitoring dashboard (deidentified) and the clinical research dashboard, which shows an exemplary behavioral anomaly where a participant did not exit their home for nearly 3 days.

### Data Analysis

Quantitative data, including participant characteristics, alert metrics, and response pathways, were summarized descriptively. Response trajectories were prospectively examined to identify alerts that elicited participant, support person, and CHW responses and those that resulted in subsequent RN action.

Qualitative data included free-text survey responses, follow-up narratives documenting health changes, and semistructured exit interviews. The data were analyzed using qualitative descriptive methods [50] and reflexive thematic analysis [51]. Coding was inductive [52] and iterative, with repeated review of written text-based data to refine codes, categories, and themes.

To strengthen rigor, the analytic team included RNs, a family nurse practitioner, bilingual CHWs, and engineering or computing expertise selected to reflect the linguistic, cultural, and clinical heterogeneity of the study population. The team met across 4 synchronous analytic circles and 8 asynchronous review sessions to discuss emerging patterns, compare coding perspectives, and refine thematic structure. After each session, written analytic summaries were synthesized into a shared memo for iterative team review. Related subthemes were consolidated into higher-order concepts through consensus discussion. An audit trail of analytic decisions, supporting written text-based excerpts, and reflexive memos was maintained throughout to enhance transparency and cultural sensitivity.

## Results

### Participant Characteristics

**Figure 2.**
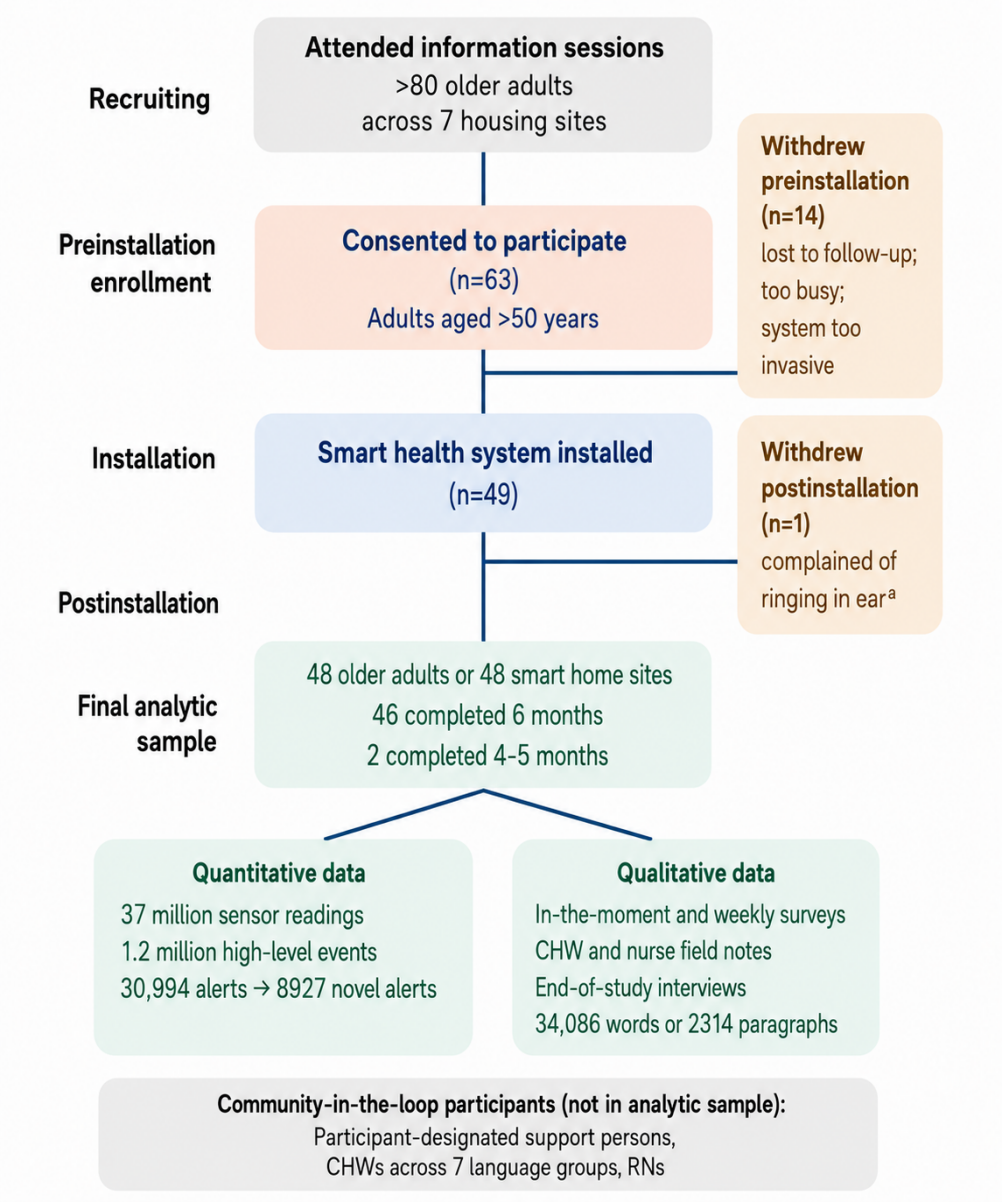
Participant flow through the smart health system study. From >80 older adults who attended initial information sessions across 7 subsidized housing sites and individual low-income constituent homes of community partners, 63 consented to participate. Following 14 preinstallation withdrawals and 1 postinstallation withdrawal, the final analytic sample consisted of 48 older adults (48 smart home sites), of whom 46 completed the full 6-month monitoring period and 2 completed 4 to 5 months (≥2/3 of the study duration). ^a^The ambient sensors did not have sound-producing capabilities. CHW: community health worker; RN: registered nurse.

A total of 63 older adults consented to participate in the study, of whom 46 completed 6 months of monitoring and 2 completed 4 and 5 months of monitoring, respectively. These 2 individuals were included in the total sample because they completed two-thirds or more of the study. Fifteen participants withdrew, with reasons including privacy and system invasiveness, discomfort with the perceived invasiveness of questions asked by the Internet Service Provider if the internet was not already connected in the home (free internet was available in subsidized housing), and time constraints. The total sample included 48 older adults (ie, 48 smart home sites). Figure 2 summarizes participant flow from initial information sessions through to the final analytic sample.

Sample demographics are presented in Table 2. Participants ranged in age from 54 to 94 years, with 77.1% (n=37) identifying as female. Educational attainment varied, with 44% (n=18) holding a high school diploma and 17% a college degree. Seven languages were represented across 4 racial groups and 8 ethnicities. Most participants lived alone (n=44, 92%) and had access to the internet before consenting to participate (n=38, 79%). Ten (21%) participants received internet access via study-provided hotspots. The most prevalent conditions among participants were heart (n=23, 47.9%), musculoskeletal (n=12, 25%), and gastrointestinal (n=6, 12.5%) conditions.

**Table 2.** Sample demographics (N=48).

Demographic variables	Participants
Age (y), mean (SD; median, IQR; range)	76.1 (8.3; 80.5, 72-83; 54‐94)
Total (one missing response)	47
Education level, n (%)	
Some middle school or no degree	2 (4.9)
Some high school or no diploma	3 (7.3)
High school or General Educational Development	18 (43.9)
Some college or no degree	11 (26.8)
Associate degree	5 (12.2)
Bachelor’s degree	2 (4.9)
Total	41 (100)
Sex, n (%)	
Female	37 (77.1)
Male	11 (22.9)
Total	48 (100)
Race ethnicity (language), n (%)	
Asian Chinese (Cantonese)	5 (10.4)
Asian Chinese (Mandarin)	3 (6.3)
Asian Korean	2 (4.2)
Asian Vietnamese	6 (12.5)
Asian	1 (2.1)
African American	1 (2.1)
Native American—Indian	1 (2.1)
White (Russian)	1 (2.1)
White (English)	28 (58.3)
Total	48 (100.1^a^)
Living arrangement, n (%)	
Independent (lives alone)	44 (91.7)
Lives with family	4 (8.3)
Total	48 (100)
Internet, n (%)	
Already had internet	38 (79.2)
Internet provided (hotspot)	10 (20.8)
Total	48 (100)
Chronic conditions, n	
Eyes-ears-nose-throat (sinusitis, vertigo, glaucoma)	3
Brain and mental health (traumatic brain injury, cerebrovascular disease, sleep apnea, panic disorder, bipolar disorder, posttraumatic stress disorder)	5
Metabolic-hormone (type 2 diabetes, hypothyroidism, gout)	14
Heart (congestive heart failure, coronary artery disease, hypertension, high cholesterol)	23
Lung (chronic obstructive pulmonary disorder, asthma)	7
Gastrointestinal (irritable bowel, acid reflux)	6
Musculoskeletal (arthritis, osteoarthritis, carpal tunnel, restless leg syndrome, chronic back pain, osteoporosis)	12
Cancer	1

a The sum of percentages could exceed 100 due to rounding.

### Alert Response Rates and Follow-Up Actions

Participants received 4719 emails and SMS text message alert reminders when baseline movement patterns changed, breaching thresholds of the custom rules-based monitoring algorithm. If a participant did not respond to their email alert within an hour, they received an SMS text message reminder. If they still did not respond, their support person received an email followed by an SMS text message alert. If the support person did not respond, the alert was automatically escalated to a CHW who then contacted the participant as soon as possible by phone. After making contact, the CHW responded to the alert on behalf of the participant and either documented a nonissue or supported the health situation as needed, including escalating a health issue to the research team RN as needed per protocol. CHW and RN responses to alerts included phone outreach and asking targeted questions related to 1 of 6 alert types (Homebody, Daytime Resting, Eating Routines, Bathing, Restless Night, Changing Sleep Routines). For example, for a Homebody alert, the participant would be asked how they were feeling and how long it had been since they last left their house or had visitors over. For a Restless Night alert, the participant would be asked about changes in bedtime, morning wake time, and the number of sleep interruptions that included getting out of bed, as well as the participant’s perspective on the cause of sleep interruptions (eg, needing to go to the bathroom to void, experiencing pain, or worrying). If appropriate, the participant was asked to take their vital signs on equipment provided by the study team. Across the 6-month monitoring period, CHWs and nurses completed 1060 participant contacts, resulting in 72 CHW or RN interventions combined (nonurgent and urgent). Commonly, CHW interventions included therapeutic listening and recommendations to avoid dehydration and get enough rest. Common RN interventions included advice to take medications as prescribed, check blood sugar, take vital signs (blood pressure, heart rate, oxygen saturation), or call their support person. For 93% (4389/4719) of the alerts, no action was required. In six instances, the RN suggested the participant call their provider the same day (n=6, <1%). In 5 instances (n=4, <1%), the RN recommended urgent care.

Participant digital response rates were 1.57% (n=74) for initial email alerts and 7.79% (n=368) for follow-up SMS text message alerts. Support persons responded to 2.37% (n=112) of the email alerts and 5.21% (n=246) of the SMS text message alerts. CHWs responded to 78.36% (n=3698) of the alerts, including 4.68% (n=221) within 1 hour, with the remaining responses occurring after 5 hours. In contrast to the very low response rates via email (n=186, 3.94%) and SMS text message (n=614, 13.01%) from participants and support persons, CHWs demonstrated substantially higher engagement, responding to 78.26% (n=3698) of the alerts.

### Net Promoter Score

Participants in the study completed a likelihood-to-recommend rating during their end-of-study interview using a 0‐10 scale (0=would never recommend the health monitoring system and 10=would highly recommend it to family or friends). Fifteen participants did not provide a rating. One response knowingly exceeded the scale maximum and was recoded to 10 prior to analysis in accordance with the instrument’s scale limits.

Among respondents (n=33), the mean likelihood-to-recommend rating was 8.68 (SD 1.68), the median was 9.0 (IQR 8.0‐10.0), and scores ranged from 3 to 10.

Following a standard Net Promoter Score methodology, respondents were then classified as promoters (9-10), passives (7-8), or detractors (0‐6). Most respondents were promoters (22/33, 67%), while 21% (n=7) were passives and 12% (n=4) were detractors, resulting in a Net Promoter Score of +55.

### Qualitative Insights From Participant Narratives

#### Overview

Three major themes emerged from qualitative analysis—Alone, Trust, and Human Connection—encompassing 11 subthemes and related supporting concepts (Table 3). Narrative data were drawn from in-the-moment surveys with free-text fields, CHW and RN field notes, and end-of-study interviews. The corpus included 34,086 words across 2314 paragraphs. Multimedia Appendix 1 contains an extended table of themes and supporting quotations from participants’ own words.

**Table 3.** Major themes, subthemes, and supporting concepts emerging from text-based data written by participants, community health workers (CHWs), and registered nurses (RNs).

Theme	Subthemes	Supporting concepts
Alone	Safety, Timing, Reassurance	Watched Over, Comfort, Loneliness, Spoken Language, Promise
Trust	Safe or Secure, Harm, Burden	Fraud, Data Ownership, Reliance, Normalizing Symptoms
Human Connection	Features, Personalization, Digital Distress	Expectations, AI Not Good at Context, Who Assesses Symptoms

#### Theme 1: Alone

Participants frequently described concerns about being alone while managing multiple chronic conditions, particularly when feeling unwell or lacking nearby family support. Many described the system as increasing feelings of safety, support, and connection. Several participants stated that the system would be especially valuable for those living alone or without family support. One participant shared that at “[my] age and being alone it made [me] feel safe and supported” (participant 25), while another described seeing the sensor light activate at night and thinking, “I’m not alone” (participant 1).

Timing also emerged within this theme, with some participants describing the system as more relevant during periods of worsening illness or future decline. Participants additionally described the comfort associated with the system’s presence in the home.

CHW and RN narratives documented many cases in which participants preferred direct phone communication rather than email or SMS text message alerts. Participants were much more responsive to calls than emails or SMS text messages. In addition, they were more responsive when the calls were conducted in their preferred language.

#### Theme 2: Trust

Trust-related narratives centered on technology, the clinical team introducing the system, data privacy, fraud, and system reliability. Some participants also described broader distrust of the health care system. One participant stated:

*I’ve kind of given up on seeing doctors for it [neuropathy pain]; they don’t seem to do much or offer any real help.*

[Participant 50]

At the same time, participants described trust in nurses and reassurance associated with nurse availability. One participant noted it was comforting to have “a nurse more available than to wait on the line for a nurse” (participant 35). Others described reassurance for distant family members and reduced caregiving pressure, noting that the system allowed loved ones to know they were being “watched over” (participant 23) and the system could “take pressure off [my] daughter” (participant 20).

Three recurring subthemes were evident: Trust in Nurses, Data Security and Identity Protection, and Cultural Values surrounding intergenerational caregiving. Participants frequently reported answering calls only from known phone numbers, and several added the research number to their phone contacts to avoid thinking the number was a scam caller. Among culturally based communities, participants also questioned how the system aligned with traditional family caregiving expectations.

A cross-cutting concept involved participants’ own perceptions of health in relation to the system’s sensor-based feedback. One participant described ignoring alerts after perceiving that the system had misinterpreted movements (participant 56), whereas another interpreted the absence of an alert as an indication that she was well despite recent symptoms (participant 57).

#### Theme 3: Human Connection

Participants consistently described preferring direct contact with CHWs or nurses, by phone or in person, over automated alerts and response systems. Many requested CHW or RN phone numbers to save in their contacts and described these interactions as the most valued component of the system.

Access to a health care team member was also described in relation to contextual personalization when system alerts did not align with individual routines. One participant explained:

*[M]y pattern is different from others, and the system was not getting it. I kept getting alerts that were not relevant to me … but if it worked, I would have a direct nurse to call versus an on-call nurse.*

[Participant 47]

Participants also described uncertainty about whether they were using the system correctly, frequently asking questions such as “am I doing it right?” (participant 1). Others reported discomfort with sensor activity, including flashing lights that were “sometimes … bothersome” (participant 11).

Persons withdrawing from the study, both before and after the installation of the sensor, reported concerns about the lack of personalization, invasion of privacy, lack of preferred-language communication, and uncertainty regarding whether a human or the technology was responsible for monitoring health changes.

## Discussion

### Overview

#### Central Findings

This mixed methods study extends prior smart home and aging-in-place research by centering older adults managing multiple chronic conditions while experiencing poverty. Consistent with prior work, trust, avoiding aloneness, and human connection emerged as foundational to adoption [12,34,41,43,53-55]. Our findings deepen this literature by showing that adoption barriers in low-income settings extend beyond usability and technical performance to include identity theft concerns, digital distress, cultural caregiving expectations, and mistrust of institutional systems.

A central finding was that trust operated at multiple levels: trust in the technology itself, trust in the people introducing and supporting it, and trust in one’s own bodily awareness relative to system feedback. Participants’ willingness to engage was strongly shaped by the presence of trusted RNs and CHWs, who functioned as relational anchors and trusted messengers. This aligns with prior research showing that older adults prefer health technologies that augment, rather than replace, human care [56-58]. In this study, RN and CHW involvement increased confidence in the system, reduced uncertainty, and helped participants contextualize alerts within daily life.

The Human Connection theme further reinforces that relational support is not merely an implementation enhancement but may be a core design requirement for aging-in-place technologies. Participants consistently preferred speaking with CHWs or RNs over interacting with automated email or SMS text message alerts, despite generally favorable overall system ratings. High CHW response rates, alongside low participant digital response rates, suggest that human-mediated communication may be more acceptable than direct self-management of digital alerts, particularly among older adults navigating multimorbidity and poverty. These findings support the integration of human-centered communication pathways into smart home ecosystems, ensuring that technology serves as an extension of trusted care relationships rather than a replacement. Collectively, these findings (the need for human connection alongside low digital response rates) argue against a binary choice between fully automated and fully human-mediated monitoring. Hybrid, tiered models may offer a more pragmatic path forward. Automated sensing can support continuous objective data capture, while human contact, particularly by phone, remains the primary mode of communicating clinically relevant changes. Such a model could reserve intensive CHW and RN involvement for higher-acuity alerts or higher-risk participants. Lower-acuity changes could be routed for brief check-ins by support persons or CHWs or asynchronous follow-up, allowing the relational core of the system to scale without requiring proportional growth in staffing.

#### Digital Distress

The findings also introduce digital distress as an important extension of the broader technostress literature. The first clues that the concept of digital distress would eventually emerge from the data were noted during the installation phase. Seven people changed their minds about participating after encountering too many stress-inducing personal and financial questions from internet service providers when enrolling in free internet so they could participate in the study. (Note: this led to a design change where we added hot spots to the system.) Additionally, 1 person said it would be too stressful to receive calls from unknown numbers (ie, for the nursing calls), and another said the system was too invasive and would stress them out. Overall, digital distress was noted when technologies felt intrusive, difficult to interpret, poorly personalized, or disconnected from participants’ language and cultural context. Digital stress was especially pronounced when users were uncertain whether a human or the system was responsible for monitoring health changes. This role ambiguity, combined with concerns about fraud, flashing sensors, or burdensome connectivity processes, contributed to participant discomfort and study withdrawal. For older adults experiencing poverty, digital distress may be amplified by chronic illness burden, financial precarity, and prior experiences with scams or identity theft, making low-burden and transparent workflows essential. The antidote is the *human connection*, which can mitigate stress, reduce *aloneness*, and promote *trust*.

#### Safety

A related safety implication concerns the risk of false reassurance through error of omission. Though participants were told that the absence of alerts does not mean the health is stable, some participants still interpreted the absence of alerts as evidence that they were well, even when experiencing symptoms. This suggests that missed detections or insufficient personalization may unintentionally increase reliance on incomplete system feedback. Future smart home systems should therefore prioritize explainable alert logic, transparent thresholds, and explicit communication that the absence of an alert does not equate to clinical stability. These findings have direct implications for digital safety, implementation ethics, and the design of AI-supported aging-in-place tools.

#### Older Adults’ Resources

Poverty-specific barriers were highly influential. The process of accessing free internet felt invasive or financially risky and resulted in several withdrawals from this study. Fear of hidden fees, identity theft, and lack of ongoing support after study completion undermined the willingness to continue with the internet sign-up process. These findings highlight how connectivity itself can become a structural barrier, even when technologies are designed to be low cost.

Though we met our target of less than US $400 per home for hardware, there were other development and implementation costs; primarily personnel time (engineering contractor, nursing, multilingual CHWs, research assistants), training staff, and hotspot connectivity subscriptions for participants without internet. Technical assistance throughout the study was included in the engineering contract, but that may not always be the case. Often, technical assistance is charged separately. Multilingual CHWs received differential pay for each additional language spoken (an additional US $3 per hour). Supply chain issues and inflation impacted hardware availability and costs over a 6-month purchase timeframe during the COVID-19 pandemic (November 2021-May 2022). These pragmatic discoveries suggest that equitable scaling requires budgeting not only for hardware but also for connectivity, technical maintenance, and language access.

### Feasibility

#### Built Environment

The built environment also shaped feasibility. Older housing, limited outlets, and brick-and-steel walls interfered with connectivity and sensor placement. Participants also disliked visible flashing lights and preferred less obtrusive hardware. These findings reinforce that environmental adaptability and unobtrusive sensor design are central to adoption, especially in subsidized or aging housing where infrastructure limitations are common.

#### Individualization

Our results further suggest that rules-based algorithms are insufficient for diverse older adult populations. Participants frequently reported irrelevant alerts or missed meaningful changes because standardized thresholds failed to reflect individualized routines. One participant responded to an email alert saying they wanted alerts to their support person to stop because “I don’t want to alarm her!” and the alerts, in the participant’s opinion, were inaccurate. This mismatch undermined trust and increased burden. Context-aware machine learning models that adapt to personal schedules, cultural practices, and evolving health patterns may offer a more appropriate pathway for equitable smart home monitoring. However, such systems must preserve transparency and human oversight to avoid exacerbating mistrust.

#### Cultural Sensitivity

Cultural expectations around caregiving were another key determinant of adoption. In some communities, participants expressed concern that technology might undermine family caregiving roles or conflict with intergenerational obligations. Systems that intentionally incorporate family, CHWs, and RN oversight may therefore better align with users’ values and improve engagement. Rather than positioning monitoring technologies as substitutes for caregiving, framing them as tools that strengthen family and community support structures may enhance trust and long-term use.

These contextual findings suggest that successful smart home health systems for older adults experiencing poverty require more technical accuracy than was possible within the scope of this study. Such systems require relational trust, cultural responsiveness, personalization, environmental adaptability, and sustainable implementation infrastructure. Human support—particularly through CHWs and nurses—appears central to mitigating digital distress, strengthening trust, and promoting equitable aging in place.

### Principal Results

This study identified several barriers to smart home adoption among older adults managing multiple chronic conditions while experiencing poverty. Beyond technical feasibility, adoption was shaped by digital access barriers, identity theft concerns, cultural caregiving expectations, environmental constraints, and structural inequities tied to poverty. Across qualitative themes, Aloneness, Trust, and Human Connection emerged as central determinants of engagement.

A key finding was that participants consistently preferred human-mediated communication, particularly with CHWs and RNs, over automated alerts. Although direct digital response rates were low, CHW engagement was high, suggesting that integrating trusted community and clinical personnel into the health care-technology-patient loop may be critical for sustained use in low-income settings. Future research should test how to reduce the staffing intensity of this model while preserving the human contact participants valued, including through acuity-based tiering, machine learning personalization, and language-concordant outreach.

This study also introduces digital distress, a novel concept describing stress that arises when technology feels intrusive, confusing, insufficiently personalized, or disconnected from linguistic and cultural context. Digital distress was most evident when participants were uncertain how the system worked, whether they were using it correctly, or who was responsible for monitoring health changes.

Collectively, these findings indicate that smart health systems designed around human relationships, cultural responsiveness, and contextual adaptation, and implemented with trusted CHW and nursing support, may better promote equitable aging in place among older adults managing multimorbidity while experiencing poverty.

### Limitations and Future Research

This study has several limitations. The sample was relatively small and drawn from a specific population of older adults managing multimorbidity while experiencing poverty. Despite the extant literature defining older adulthood as age 65 years or above, the study sample included immigrant adults aged 50 years or above who were managing multimorbidity, which commonly affects these populations at younger ages due to the impacts of social determinants of health. This may limit transferability across populations, settings, and cultural contexts. In addition, the smart health system was a prototype that underwent iterative refinement during the study, and its rules-based alerting approach may have contributed to irrelevant or missed alerts. The intensive involvement of CHWs and RNs, while central to equitable implementation, may also limit scalability in other care settings. Because digital distress emerged inductively within this context, additional research is needed to examine its relevance across populations and technologies. Future work should evaluate scalable machine learning approaches, multilingual and culturally responsive interfaces, improved alert accuracy and data visualization, integration with clinical workflows, and strategies for communicating automated messages and engaging older adults and families with sensor-derived health data while preserving simplicity and trusted human support.

### Conclusions

Smart home health monitoring systems hold promise for supporting aging in place and helping older adults maintain independence, but their success among those experiencing poverty depends on more than technological innovation. If systems are not designed with attention to the unique barriers faced by this population, they risk reinforcing existing health inequities. This study demonstrates that adoption is shaped by a complex interplay of digital literacy, cultural values, trust in institutions, and the physical realities of low-income housing. A community-in-the-loop approach that integrates ambient sensors, automated unobtrusive monitoring, and human-centered care networks may reduce isolation, foster trust, and maintain human connection while offering real-time health insights. To be effective and equitable, future smart health systems must be co-designed with communities and prioritize personalization, cultural sensitivity, and low-burden engagement. In addition, technology-enabled health monitoring for the home must include intentional, explicit strategies to mitigate digital distress. These findings offer a roadmap for researchers, designers, and policymakers seeking to close the digital divide and promote health equity through technology-enabled care.

## Supplementary material

10.2196/88290 Multimedia Appendix 1 Smart health system prototype description (mHealth Evidence Reporting and Assessment aligned).

## Abbreviations

CART: collaborative aging research using technology
CASAS: Center for Advanced Studies in Adaptive Systems
CHW: community health worker
IoT: Internet of Things
IRB: Institutional Review Board
PI: primary investigator
RN: registered nurse
