# Supplementary material for: A Community-in-the-Loop Approach to Smart Home Monitoring for Aging in Place: Mixed Methods Evaluation of a Co-Designed Prototype
Source: JMIR Aging. 2026 Jul 23;9:e88290. doi: 10.2196/88290 (PMC13395425; doi:10.2196/88290)
Supplement: Multimedia Appendix 1 [file aging-v9-e88290-s001.docx]

# Multimedia Appendix 1. Smart Health System Prototype Description (mERA-Aligned)

Detailed information about the smart health system (SHS) prototype is presented using the mHealth Evidence Reporting and Assessment (mERA) framework^1^ to support transparent reporting of the system’s technical, workflow, and implementation components. This structure was selected to guide reporting here because the prototype incorporated a mobile health communication layer through SMS text message alert escalation, and because the study evaluated system feasibility and adoptability in real-world residential environments rather than controlled laboratory settings. Applying mERA when reporting the prototype’s technical features strengthens reporting of contextual factors—such as connectivity, housing infrastructure, cultural adaptation, workflow integration, and human support—that are critical when translating smart home health monitoring proof-of-concept research into complex naturalistic settings.

## 1. Infrastructure

The SHS prototype was a sensor-based, in-home monitoring platform designed to detect changes in daily routines among older adults aging in place. The system combined ambient sensing, rules-based analytics, and community-in-the-loop clinical workflows to support early identification of potential health changes. The prototype was deployed for 6-months in 48 participant unique homes between February 2022 and April 2024. Across the study period, the system collected >37 million raw sensor readings, which were processed into approximately 1.2 million high-level behavioral events (e.g., “at location”).

Homes were primarily older subsidized housing units with variable electrical access, limited outlets, and building materials that sometimes interfered with wireless connectivity. For participants without home internet, mobile hotspots were provided, typically positioned near windows to optimize signal strength. These infrastructure requirements were critical to system operation and influenced deployment cost and reliability.

## 2. Technology Platform

Each home installation included:

- 1 Raspberry Pi 4 Model B
- 1 Z-Wave USB controller (Z-stick)
- Up to 6 passive infrared (PIR) motion sensors
- Up to 2 magnetic door sensors

Most homes used 4 motion sensors and 2 door sensors (main entry door, refrigerator door). Sensors were labeled by room (i.e., bedroom, bathroom, kitchen, living room) and installed with participant input to optimize acceptability and motion detection (Figure A1). Devices were managed using openHAB (Open Home Automation Bus), an open-source home automation platform, and securely synchronized with the ASSETs™ platform hosted by Hekademeia™.^2^

**Figure A1.** The devices comprising the SHS include a Raspberry Pi (keyboard) with Z-stick (attached to keyboard; for connectivity between sensors and Raspberry Pi), power cord (attached to keyboard), up to 6 motion multi-sensors [passive infrared, humidity, light]: 2 round, 2 square) and 2 magnetic door use sensors (rectangle set).


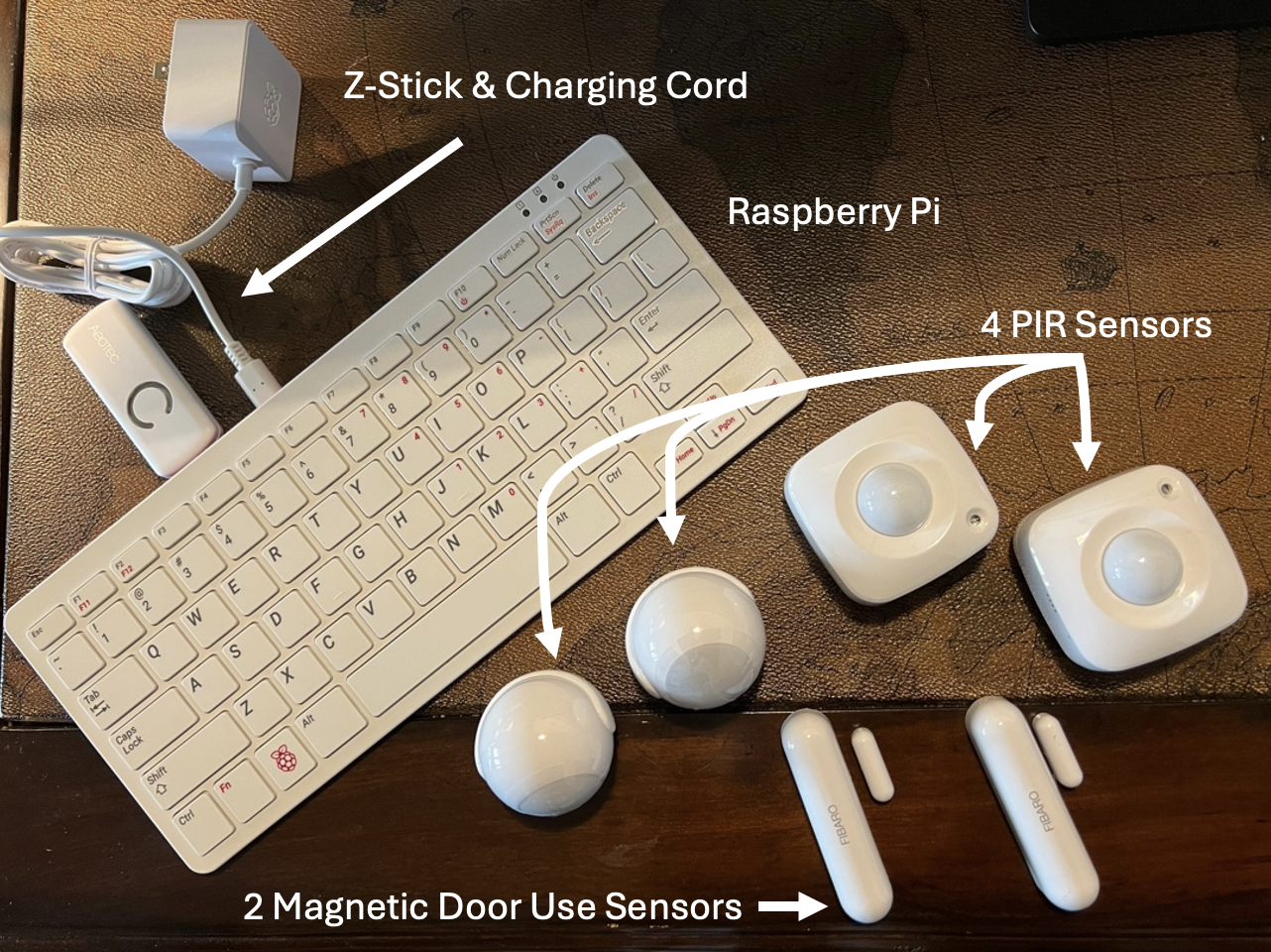


The in-home devices were configured to send sensor data every 1-3 hours. Every 5 minutes, the Raspberry Pi gathered its local IP, WAN IP, and two VPN network IP addresses, transmitting this information to ASSETs servers. During synchronization, the device sent all sensor data collected since the last successful sync. Processing systems were designed to backfill data gaps in case of synchronization failures. Real-time connectivity status was determined by whether the device synchronized within the expected interval. Persistent signals from openHAB indicated sensor connectivity issues, while a lack of synchronization attempts signaled internet connectivity problems.

A custom rules-based algorithm designed specifically for the study processed sensor data to detect clinically relevant changes in daily routines (e.g., decreased mobility, altered sleep, missed meals). Raw sensor readings were aggregated into higher-level behavioral events. For example, multiple motion detections could be consolidated into a single “location occupancy” event. These processed events formed the basis for downstream alert generation.

Two dashboards were developed: one that was simplified for participants and their support persons, one detailed for the clinical research team. Both dashboards supported the community-in-the-loop interpretation of system data. The participant dashboard displayed written text-based summaries of movement trends and alerts, whereas the research dashboard provided detailed temporal views, connectivity monitoring, and anomaly review tools. The participant dashboard was designed to reduce cognitive burden (Figure A2). The clinical research team dashboard included a detailed visualization of movement patterns over time, alert frequency, connectivity status, and ‘zoom’ functionality for reviewing anomalies (Figure A3).

**Figure A2.** Health-monitoring dashboard with identifiers removed.


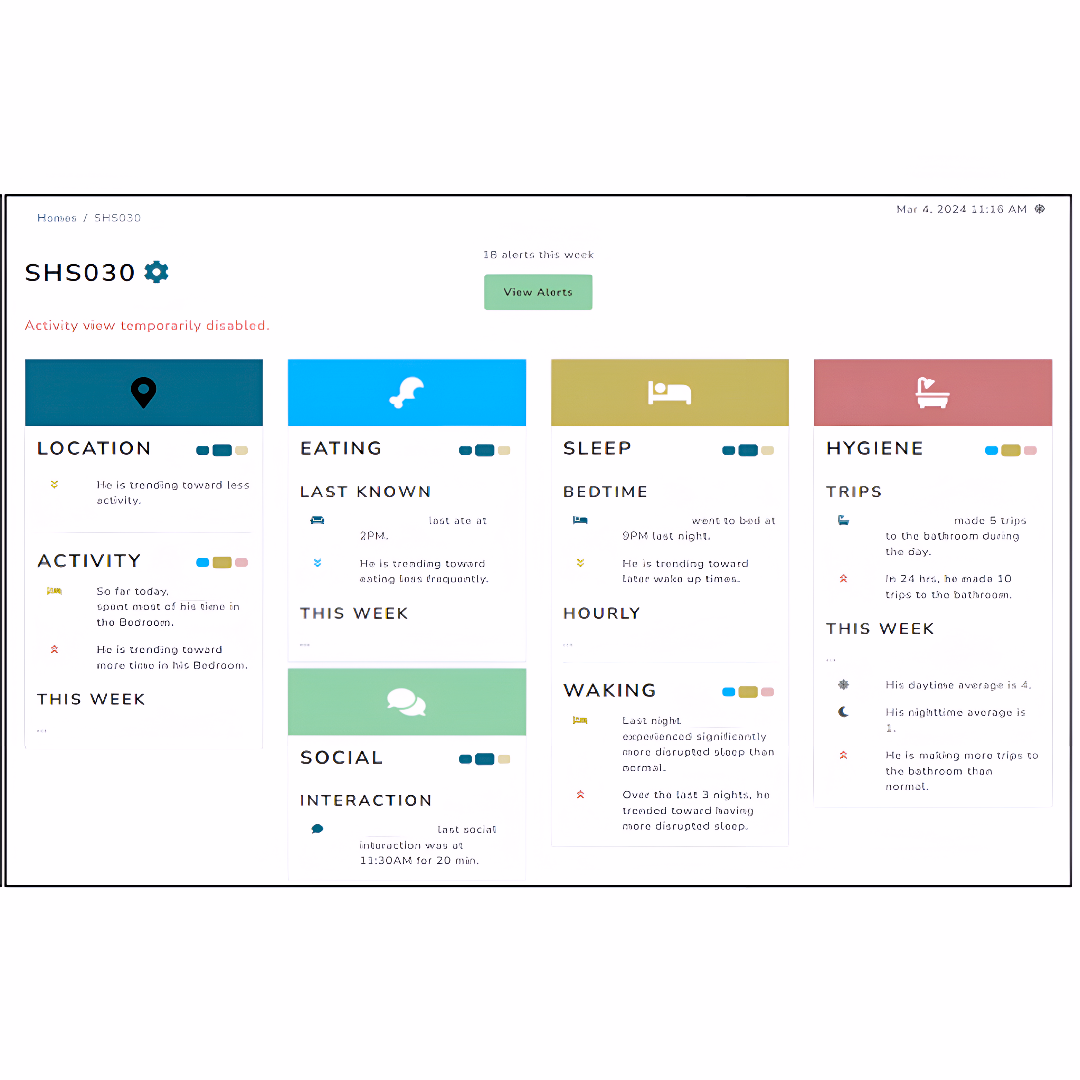


**Figure A3.** Clinical research dashboard showing a behavior anomaly inside the red circle where a participant did not exit their home for nearly 3 days.


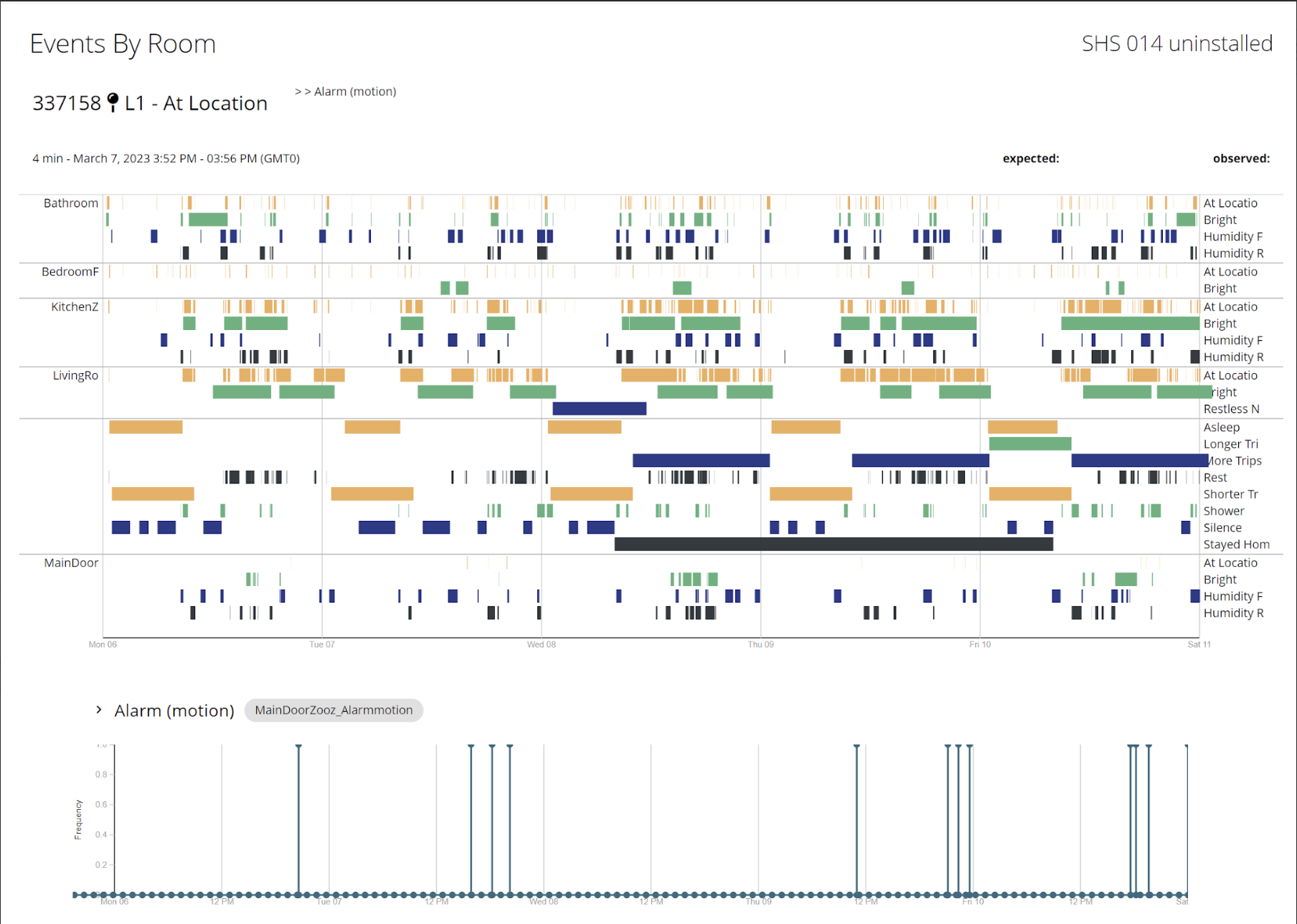


Alerts were iteratively adjusted over time until a pragmatic sensitivity level was achieved; defined as participants were not receiving more than 1 of the same type of alert per week and no more than 3 alerts total per week. To achieve this goal, alerts generated for the same type of information (e.g., sleep) were clustered together into a single alert that was sent to the participant (e.g., Restless Nights).

Alerts were triggered when predefined thresholds were exceeded that represented deviation in routine behaviors possibly representing a change in health status (i.e., symptoms), including changes in mobility patterns, sleep behaviors, kitchen activity, bathroom usage, and home entry/exit patterns (Table A1). Sensor-derived activity patterns were associated with real-world health events, including reduced movement during illness episodes, increased nighttime activity during acute conditions, and changes in mobility during flare-ups or recovery periods. These observations informed alert refinement and clinical follow-up processes.

**Table A1.** Alert categories associated with abnormal movement patterns and descriptions for criteria encoded in the algorithm.

| **Alert Name**  **(Participant)** | **Alert Name (Nurse)** | **Description** |
| --- | --- | --- |
| Homebody | Social Isolation | Main door not used for >48 hours and motion detected inside the home. |
| Daytime Resting | Decreased Activities | Variety of at location sensor readings decrease by >30% or bed location increases by >30% in 24 hours. |
| Eating Routines | Kitchen Use | A 50% increase or decrease in time spent in the kitchen. |
| Bathing | Decreased Hygiene | No humidity-up in bathroom detected for >3 days. Number of days set per participant reported routine. |
| Restless Night | Sleep Disturbance | Location other than bed for >4 hours at night. Nighttime beginning and ending hours set per participant reported routine. |
| Changing Sleep Routines | Sleep Patterns | Going to bed earlier or later than normal by >2 hours. Normal bedtime and awake time specified uniquely per participant. |

## 3. Interoperability and Clinical Workflow Context

The prototype was designed as a community-in-the-loop monitoring system rather than a stand-alone alerting tool. Sensor-derived alerts were integrated into a simulated structured clinical workflow involving participant notification, designated family/support person notification, community health worker (CHW) follow-up, and RN escalation when indicated.

This workflow allowed sensor data to support community-based care coordination and nursing assessment, functioning as a supplemental monitoring layer within existing healthcare relationships. When an alert was triggered, participants first received an email and SMS message prompting them to respond with “I’m okay” or “I’m not okay” via a secure survey link. If unacknowledged, the alert escalated to the participant’s designated support person (family or friend). It was escalated again, if unacknowledged, to a trained CHW. CHWs contacted participants by phone, assessed health status by talking to the participant and asking laymen’s questions to elicit information about how the participant was physically or mentally feeling. When needed, CHWs escalated concerns to an RN. Due to research resource constraints, notifications were sent during business hours only so they could be responded to in a timely manner. Participants were notified of this during the consenting process.

## 4. Real-World Prototype Trial of Intervention Delivery

No intervention was delivered and no effect was measured. However, the prototype continuously monitored ambient movement, sleep-related location changes, door use, kitchen activity, and humidity-associated bathing routines. The rules-based algorithm generated alerts when deviations from individualized baselines exceeded predefined thresholds. Alerts were delivered through email, SMS text message, and phone-based follow-up during business hours only (for the purposes of research). Similar alerts within 7 days were combined into a single alert to reduce alert fatigue. For example, an anomaly regarding ‘increased bathroom use’ on the same night of Restless Night alert were combined into one alert requiring one survey response. Across deployment, 37 million sensor readings were generated yielding 4719 novel alerts after combining similar alerts.

## 5. Prototype Real-World Trial Intervention Content

The real-world simulated intervention combined:

- passive ambient sensing
- participant-facing alert messages
- secure in-the-moment survey links
- CHW phone outreach
- RN review when clinically indicated
- participant and research dashboards

## 6. Usability Testing and User Feedback

System configuration and sensor placement were co-designed with participants. Participants and research staff jointly chose each sensor locations. The system incorporated co-designed features to support multi-resident and multi-generational households, cultural caregiving norms, and privacy preferences. For example, family members could select which sensors were deactivated when family members were present and which were activated for expanded monitoring when the older adult was alone. Remote check-ins by family members were possible through the participant-facing dashboard. These features were designed to balance safety, privacy, and cultural expectations. Qualitative feedback informed iterative refinement of alert thresholds and clustering rules to reduce burden. Final qualitative and quantitative findings indicated that older adults with multimorbidity who were also experiencing poverty were open to monitoring through a system like the one prototyped and tested in this study. However, adoption depended on technical performance, trust in both the technology and the people associated with it, and cultural sensitivity.

## 7. Access and Participant Entry

Participants entered the program through in-home installation visits. For those lacking internet access, the study team facilitated hotspot deployment or assistance with subsidized internet enrollment. Entry barriers included technology discomfort, concerns about hidden fees, and identity theft fears related to internet enrollment workflows. Entry facilitators included the participant added a CHW or RN’s phone number to their mobile device contacts and communicating with participants in their preferred language.

Though free internet was available to subsidized housing residents through the federal government’s Affordable Connectivity Program during the study period (2022-2024), signing up for the service involved needing to provide the Internet Service Provider with bank or debit or credit card information plus other personal information that caused residents to fear fees, identity theft, and more. All participants who consented to the study and started the process of obtaining free internet ultimately reversed their decision on both aspects (obtaining internet and participating in the study). Smart home monitoring systems now and in the future should include internet connections as part of the provided system.

## 8. Cost Assessment

The initial target cost was <$400 per home, but real-world deployment costs approached $800 per home over 6 months due to inflation, mobile hotspots, multilingual support needs, troubleshooting visits, sensor replacement, and technical maintenance. These findings are important for scalability.

## 9. Contextual Adaptability

The SHS incorporated contextual and culturally responsive adaptations, included:

- selective room monitoring in multigenerational households
- family-defined privacy zones
- time-based expansion of monitoring when the older adult was alone
- remote reassurance features for family caregivers
- verbal communication in participants’ preferred language

These features supported alignment with caregiving norms, privacy preferences, and daily routines. Future contextual and cultural adaptability should also include written and dashboard displays that accommodate additional languages.

## 10. Data Security and Privacy

Sensor data were securely transmitted through VPN-enabled synchronization to the ASSETs™ servers. Devices transmitted:

- local IP
- WAN IP
- VPN network IPs
- buffered sensor events since last sync

Participants were informed that the smart home system was a research prototype, not an emergency detection system, and were instructed to continue using routine healthcare and emergency services.

## 11. Fidelity and Technical Performance

Technical fidelity challenges included thick walls, limited outlets, hotspot instability, and sensor visibility (e.g., flashing lights), all of which informed later refinements, some of which were not overcome during the study period. For example, although the product information for the round sensors indicated that the lights could be turned off, they could only be dimmed rather than completely turned off. Some participants reported liking the sensor light turning on when they walked by while others did not like it, including one participant who withdrew from the study due to complaints about the sensors.

Fidelity was also evaluated at the level of clinical signal usefulness, focusing on whether system outputs remained sufficiently accurate, interpretable, and actionable to support follow-up within the community-in-the-loop workflow. A key technical performance issue was the balance between over-alerting and under-detection as participant routines evolved over the 6-month monitoring period. Because daily patterns changed with seasonal behavior shifts, holidays, visitors and more, initially appropriate thresholds sometimes became misaligned with current routines. This reduced signal specificity and required iterative recalibration of alert rules to preserve practical usefulness.

Performance fidelity therefore depended not only on hardware uptime, but on the stability of individualized behavioral baselines over time. Homes in which routines were highly regular tended to yield more clinically interpretable alerts, whereas greater day-to-day variability increased the likelihood of low-value notifications or missed meaningful deviations. Ongoing review of alert narratives by CHWs and RNs functioned as an additional fidelity mechanism by identifying when algorithm outputs no longer aligned with lived context – generally experienced as too many alerts. These ‘human-in-the-loop’ observations informed refinement of clustering rules, threshold windows, and participant-specific expectations.

This experience suggests that fidelity in real-world smart home monitoring should be conceptualized as longitudinal algorithm-context fit—the extent to which detection logic continues to reflect changing human routines over time—rather than solely as device reliability.

## Prototype Description Summary

The prototype integrated low-cost hardware, ambient sensing, rules-based analytics, and human-centered clinical workflows supported by a community care model. The system was designed to support early detection of health changes while maintaining flexibility for diverse home environments and user needs.

# Extended Results

## Exemplar Quotations Supporting Thematic Analysis

To maintain readability in the Results section while preserving transparency, we include extended quotations here that illustrate each theme and subtheme (including Digital Distress and the cross‑cutting concept of trusting one’s own knowledge of self versus the system). Quotations are organized with participant IDs, and a one‑line context note; see the Results and Discussion for theme definitions and implementation implications.

**Appendix Table A2.** Extended exemplar quotations by theme and subtheme.

| **Theme** | **Subtheme (or Cross‑Cutting Concept)** | **Exemplar Quotation** | **Participant ID** | **Brief Context** |
| --- | --- | --- | --- | --- |
| **Alone** | Reassurance | “At my age and being alone it made me feel safe and supported.” | #25 | Feeling safer with the system while living alone. |
| **Alone** | Timing | “If I lived alone, it would be very useful.” *(echoed by several participants)* | #7; also #13, #14, #17, #25 | Value strongest when older adult is alone. |
| **Alone** | Safety | “If I was more ill, then it would give me safety.” | #15 | Utility increases with illness severity. |
| **Alone** | Watched Over | “I… came back to bed… the green light came on and I was like, I’m not alone.” | #1 | Nighttime reassurance from sensor cue. |
| **Trust** | Safe/Secure | “It was comforting to have a nurse more available than to wait on the line for a nurse…” | #35 | Trust derived from nurse accessibility. |
| **Trust** | Burden | “Let’s loved ones from far away know that their loved one is being watched over.” | #23 | Family peace of mind. |
| **Trust** | Burden | “It would take pressure off my daughter… knowing the nurse watches.” | #20 | Reducing burden on family caregiver. |
| **Trust** | Safe/Secure | “I would only answer calls from known numbers.” *(summarized sentiment)* | (Multiple) | Fraud/scam concern; known‑caller protocol needed. |
| **Trust** | **Cross‑cutting**  Harm | “The system misinterpreted my movements, so I ignored alerts.” | #56 | Prefers self‑assessment over system output. |
| **Trust** | **Cross‑cutting** Harm | “My routine has been off… I didn’t get an alert, so I thought I was good.” | #57 | Over‑reliance on absence of an alert (false reassurance). |
| **Human Connection** | Personalization; Features | “I would have a direct nurse to call versus an on‑call nurse.” | #47 | Desire for relational support during tech misfits. |
| **Human Connection** | Digital Distress | “Lights were always flashing… sometimes it was bothersome.” | #11 | Sensor visibility causing annoyance or anxiety. |
| **Human Connection** | Digital Distress | “Three participants withdrew due to discomfort with the system (intrusive, confusing, stressful).” *(Study note)* | (n=3) | Attrition attributed to digital distress. |

## Appendix 1 References

1. Agarwal S, LeFevre AE, Lee J, et al. Guidelines for Reporting of Health Interventions Using mMobile Phones: Mobile Health (mHealth) Evidence Reporting and Assessment (mERA) Checklist. *BMJ*. 2016;352:i1174. doi:10.1136/bmj.i1174

2. Hekademeia Research Solutions. Accessed November 5, 2025. https://www.hekademeia.org/
